# Can quantifying free-circulating DNA in plasma be used to identify subjects with high-grade pre-invasive endobronchial lesions?

**DOI:** 10.3892/ol.2013.1262

**Published:** 2013-03-19

**Authors:** ROBERT A.A. VAN BOERDONK, HES A.P. BROKX, PYNG LEE, CLARISSA KOOI, PIETER E. POSTMUS, PETER J.F. SNIJDERS, KATRIEN GRÜNBERG, ERIK THUNNISSEN, THOMAS G. SUTEDJA, JOHANNES M.A. DANIELS, DANIËLLE A.M. HEIDEMAN

**Affiliations:** 1 Departments of Pathology, Amsterdam 1007 MB, The Netherlands;; 2 Pulmonary Diseases, VU University Medical Center, Amsterdam 1007 MB, The Netherlands;; 3 Department of Medicine, National University Hospital, Singapore 119228, Republic of Singapore

**Keywords:** circulating plasma DNA, autofluorescence bronchoscopy, premalignant endobronchial lesion

## Abstract

Increased concentrations of free-circulating plasma DNA (cpDNA) are observed in patients with invasive cancer, including lung cancer. Whether cpDNA levels are elevated in subjects with high-grade pre-invasive lesions of lung squamous cell carcinoma (SqCC) and whether its detection may be of value for identifying subjects at the highest risk of developing lung SqCC is currently unknown. The present study assessed cpDNA levels in subjects with high- and low-grade pre-invasive squamous endobronchial lesions relative to patients with clinically overt lung SqCC and healthy controls using real-time quantitative PCR methodology. The median cpDNA levels of the patients with invasive lung SqCC (n=16) were significantly higher compared with those of the healthy controls (n=16; P<0.01), whereas the cpDNA levels in the subjects with pre-invasive lesions (n=20) did not differ from those of the controls (P=0.29). The cpDNA levels in subjects with high-grade pre-invasive lesions were highly similar to those diagnosed with low-grade pre-invasive lesions (P=0.85). Our data suggest that cpDNA levels are not increased during the pre-invasive stages of lung squamous carcinogenesis.

## Introduction

Early lung cancer detection is an important approach for reducing disease mortality rates. Low-dose spiral computed tomography (CT) scanning has demonstrated effectiveness in detecting early-stage peripheral lung cancers (1–3). However, the CT yield for early-stage central airway cancers is relatively poor (4) and its costs appear to limit wider implementation (5). Autofluorescence bronchoscopy (AFB) has been shown to be highly sensitive for the detection of early-stage (micro-) invasive carcinoma and pre-invasive lesions in the central airways (6–10). Its high false-positive rate and rather invasive approach hamper its wider use as a cost-effective screening tool (8). The detection of molecular biomarkers that are representative of the disease state in less or non-invasively collected biological fluids, such as blood or sputum, may be an alternative or addition to this imaging technique and may improve the cost-effectiveness of screening.

Analysis of free-circulating plasma DNA (cpDNA) levels is a non-invasive method that has shown the potential to detect patients with lung cancer (11–15). Significantly higher cpDNA levels have been demonstrated in patients with invasive lung cancer, independently of tumor stage, as compared with controls (13,14). However, it is currently not known whether cpDNA levels are increased in subjects with high-grade pre-invasive squamous endobronchial lesions or if cpDNA level quantification may be of value as a marker of invasive growth in these subjects. This information may contribute to our knowledge of cpDNA quantification as a potential non-invasive screening tool for identifying individuals at the highest risk of developing lung squamous cell carcinoma (SqCC). In the present study, we evaluated cpDNA levels in a cohort of subjects with AFB-visualized pre-invasive endobronchial lesions comprising the full spectrum of premalignant squamous disease, relative to patients with clinically overt lung SqCC and cancer-free, healthy controls. The current study aimed to assess whether cpDNA quantification was able to discriminate among these subgroups.

## Subjects and methods

### Study subjects

Peripheral blood was collected with informed consent from i) subjects with pre-invasive squamous endo-bronchial lesions; ii) patients diagnosed with invasive lung SqCC (stage I–IV); and iii) cancer-free, healthy individuals. Characteristics of the study population are shown in Table I. Pre-invasive endobronchial lesions were visualized by AFB in subjects at risk of lung cancer on the basis of smoking habits (>20 pack years), chronic obstructive pulmonary disease (COPD), signs/symptoms and/or history of lung or head and neck cancer, but without clinically overt lung cancer (16). For this study, the subjects were selected in such a way that the AFB-visualized lesions included the full spectrum of pre-invasive squamous endobronchial disease and that variables that may potentially confound the analysis, such as gender (P=0.49), age (P=0.14), smoking status (P=0.61), number of pack years (P=0.42), COPD status (P=0.56) and storage time of plasma specimens (P=0.19), were kept similar to those of the cancer cases. Staging of lung cancers was performed according to the 6th edition of the TNM classification system of malignant tumors (17), and histology according to the 2004 IASLC/WHO histological classification system of pre-invasive and invasive squamous lesions of the bronchus (18). This study followed the ethical guidelines of the Institutional Review Board.

### Sample handling and DNA isolation

Peripheral blood was processed within 1 h of collection. The plasma component was carefully separated by centrifuging twice at 3,000 rpm for 10 min at room temperature. The plasma specimens were stored in 1-ml aliquots at −80°C until use. DNA isolation was performed essentially as described previously (13). Briefly, DNA was isolated from a plasma aliquot using the Qiagen QIAamp DNA mini kit (Westburg, Leusden, The Netherlands) according to the manufacturer’s instructions for blood and body fluids. Specimens were spiked with plasmid DNA (pHPV16) prior to DNA isolation to control for DNA extraction efficiency.

### Plasma DNA quantification

cpDNA levels were determined in quadruplicate by means of quantitative real-time PCR amplification of the human Fra-1 gene on the ABI/Prism 7300 Real-Time PCR system (Applied BioSystems, Nieuwerkerk a/d IJssel, The Netherlands) and further quantified using a standard calibrator curve of human placental DNA (19). HPV16-specific quantitative real-time PCR to specifically quantify the plasmid DNA was also performed on the DNA isolates to assess DNA extraction efficiency (20).

### Statistical analysis

cpDNA quantification assays were performed in a blinded fashion, and afterwards the results were correlated with disease category. For analysis, the pre-invasive lesions graded as hyperplasia, squamous metaplasia, mild dysplasia and moderate dysplasia were categorized into low-grade pre-invasive disease (LGD), and severe dysplasia and carcinoma *in situ* (CIS) were categorized into high-grade pre-invasive disease (HGD). Statistical analyses were performed using the SPSS Statistics v17.0 software package (SPSS, Inc., Chicago, IL, USA). Differences in the frequencies of patient characteristics between groups were examined using Fisher’s exact and Mann-Whitney U tests. Median plasma DNA levels between groups were compared using non-parametrical Kruskal-Wallis testing.

## Results

**Figure 1:**
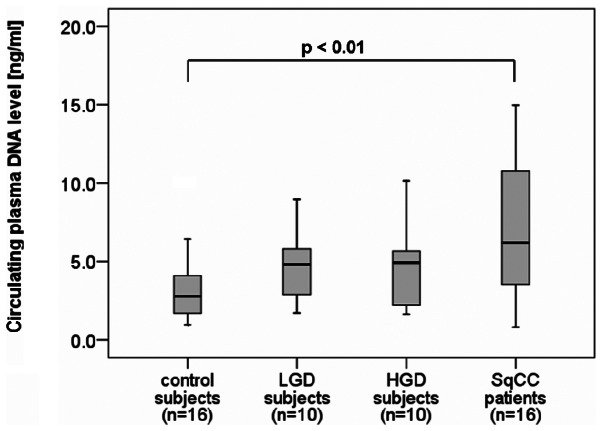
Box plot displaying cpDNA levels among cancer-free control subjects (n=16), subjects with LGD (n=10), subjects with HGD (n=10) and patients with clinically overt SqCC (n=16). Black lines within the box represent median values, and the whiskers indicate the minimum and maximum values that lie within 1.5 × interquartile range. cpDNA, free-circulating plasma DNA; LGD, low-grade pre-invasive disease; HGD, high-grade preinvasive disease; SqCC, squamous cell carcinoma.

A total of 52 plasma samples were used for comparison of cpDNA levels from i) 20 subjects with AFB-visualized preinvasive endobronchial lesions (LGD, n=10; HGD, n=10); ii) 16 patients with clinically overt, invasive SqCC (stage I, n=7; stage III, n=6; stage IV, n=3); and iii) 16 cancer-free, healthy individuals (controls). The patients with clinically overt lung cancers demonstrated significantly higher levels of cpDNA (median, 6.2 ng/ml; range, 0.8–77.6 ng/ml) as compared with the controls (P<0.01; Fig. 1). By contrast, cpDNA levels were unable to discriminate at-risk subjects with pre-invasive lesions (median, 4.9 ng/ml; range, 1.6–10.2 ng/ml) from controls (median, 2.8 ng/ml; range, 1.0–10.1 ng/ml), neither in the case of LGD (P=0.10) nor HGD (P=0.29). Moreover, the cpDNA levels in subjects who were diagnosed with HGD (median, 4.9 ng/ml; range, 1.6–10.2 ng/ml) were highly similar to those in subjects diagnosed with LGD (median, 4.8 ng/ml; range, 1.7–9.0 ng/ml; P=0.85). Of note, 3 of the 10 individuals presenting with HGD at the time of peripheral blood sampling were diagnosed with invasive lung cancer within a follow-up period of 6 months, and none of them had elevated cpDNA levels at the pre-invasive stage; their cpDNA levels were 1.6, 2.2 and 4.8 ng/ml.

## Discussion

The results of our study suggest that cpDNA levels are not increased during the pre-invasive stages of lung squamous carcinogenesis. Although cpDNA levels were significantly higher in clinically overt lung cancer, consistent with previous data (12–15), none of the subjects with pre-invasive lesions, neither LGD nor HGD, could be discriminated from controls on the basis of their cpDNA level. Our findings suggest that the quantification of cpDNA in plasma may not be a useful approach for identifying subjects with high-grade pre-invasive lesions of lung SqCC, nor for prognostication in potentially malignant conditions.

Sozzi *et al*(21,22) suggested that the release of DNA in plasma is correlated with the establishment of a relatively advanced grade of interaction between the tumor and the microenvironment. The authors demonstrated that plasma DNA levels in patients with CT-detected lung cancer, which particularly consist of small, early-stage adenocarcinomas, were comparable with those of disease-free subjects, and that only patients with clinically overt lung cancers were observed to have markedly higher levels of cpDNA. Studies on the quantification of cpDNA in subjects with precancerous lesions are scarce. Shukla *et al*(23) evaluated cpDNA levels in subjects with mucosal precancerous lesions (epithelial dysplasia) of oral squamous cell carcinoma. The authors concluded that the levels of cpDNA in subjects with oral epithelial dysplasia were not higher compared with healthy controls, which is consistent with our data of subjects with pre-invasive lesions of lung SqCC. In the study by Shukla *et al*(23), however, cpDNA levels were also not elevated in patients with oral SqCC, an observation that does not agree with our data and those of previous studies (12–15). The authors suggested this to be a property inherent to the type of neoplasm and its dissemination characteristics, which is different for lung cancer and oral cancer. The possibility that cpDNA quantification may provide a fingerprint of the aggressive behavior of different types of tumors requires further testing. This will also be of interest within screening trials in the effort to improve the clinical management of CT-detected lung cancer. Furthermore, in patients with overt cancer, the abundance of cpDNA that likely originates from the cancerous cells offers the possibility to use this source material as a non-invasive monitoring system for applying companion diagnostics to determine an appropriate lung cancer treatment, as was recently shown for the detection of epidermal growth factor receptor (EGFR) mutations for EGFR-tyrosine kinase inhibitors (24).

The strength of the current study lies in the confirmatory results of cpDNA levels in patients with squamous (pre-) cancerous lesions that well complement previous results from studies, mainly including the adenocarcinoma histotype (21,22). A limitation of this study may be the number of pre-invasive lesions included, particularly in light of the fact that only a small fraction progress to SqCC (25). However, it should be noted that the analyzed series of subjects with pre-invasive squamous endobronchial lesions form a unique study population that had only become available by close AFB surveillance of large numbers of individuals at risk of lung cancer during recent years. This reflects the important difficulties faced in studying pre-invasive endobronchial disease, given the low prevalence of high-grade lesions in asymptomatic individuals within an at-risk population and the low progression rate to SqCC (25). Collecting sufficient biomaterials representing the full spectrum of pre-invasive squamous endobronchial disease to investigate the study concept requires an extensive period of close surveillance. This underscores the uniqueness of the series examined in the present study.

In conclusion, although the median cpDNA levels of patients with invasive lung SqCC were significantly higher as compared with those of healthy controls, no elevated levels of cpDNA were observed in subjects with either LGD or HGD. cpDNA quantification does not appear to be a prognostic marker in potentially malignant conditions and is unlikely to be of value as an alternative or addition to the diagnostic algorithm of AFB for identifying patients at highest risk of developing lung cancer. Our study highlights the need to continue research efforts aiming to identify biomarkers that can be appplied to non- or less invasively collected biomaterial for the early prediction of invasive cancer in the respiratory airways to improve screening and/or diagnostic algorithms.

## Figures and Tables

**Table I:** Characteristics of study population.

Characteristic	Subjects with pre-invasive squamous endobronchial lesions	Patients diagnosed with invasive lung SqCC	Cancer-free,healthy individuals
Gender, male:female	14:6	9:7	11:5
Age (years), median (range)	62 (46–71)	63 (50–81)	56 (28–87)
COPD, yes:no:unknown	10:9:1	11:4:1	5:9:2
Smoking status, C:F:N	8:12:0	7:8:0^a^	2:4:9^a^
No. of pack years, median (range)	40 (22–120)	40 (15–60)^a^	39 (20–46)^a,b^

a Data were not available for 1 SqCC patient and 1 healthy control subject;

b never-smokers were not taken into account. SqCC, squamous cell carcinoma; COPD, chronic obstructive pulmonary disease; C, current smoker; F, former smoker; N, never smoker.
